# Supplementary material for: Genetic score associations with birthweight in preterm-born infants compared with term-born infants
Source: medRxiv. 2025 Jan 28:2025.01.21.25320880. Preprint. [Version 1] doi: 10.1101/2025.01.21.25320880 (PMC11838948; doi:10.1101/2025.01.21.25320880)
Supplement: Supplement 1 [file NIHPP2025.01.21.25320880v1-supplement-1.pdf]

medRxiv preprint doi: https://doi.org/10.1101/2025.01.21.25320880; this version posted January 28, 2025. The copyright holder for this preprint (which was not certified by peer review) is the author/funder, who has granted medRxiv a license to display the preprint in perpetuity.
It is made available under a CC-BY 4.0 International license .

Online Tables

Table 1: Demographic statistics for included samples

	Birthweight (kg; mean (sd))			Gestational duration (weeks; mean (sd))			Sex (% female)		
	All	Term	Preterm	All	Term	Preterm	All	Term	Prete
RHiNO, WALHIP, PICSI	-	-	1.53 (0.83)	-	-	30.0 (3.08)	-	-	46.
ALSPAC	3.44 (0.53)	3.49 (0.47)	2.43 (0.62)	39.5 (1.76)	39.8 (1.29)	34.3 (2.33)	49.1	49.4	42.
MCS	3.40 (0.57)	3.47 (0.49)	2.4 (0.67)	39.5 (1.94)	39.8 (1.27)	34.4 (2.24)	50.2	50.5	46.
BiB	3.41 (0.53)	3.45 (0.49)	2.46 (0.53)	39.8 (1.59)	40.0 (1.20)	35.1 (1.72)	47.4	47.6	42.

Table 2: Associations between birthweight GS and birthweight in term- and preterm-born infants. For genetic scores, “raw” indicates the score constructed using the marginal effects from the GWAS meta-analysis of the fetal genome vs own BW and “SEM” indicates the estimates from the partitioned fetal effects of the GWAS variants.

Gestation	Sex	Genetic Score	Beta	SE	P
34<=GA<37	all	raw	0.9781	0.2259	1.50E-05
34<=GA<37	all	SEM	1.0047	0.231	1.36E-05
32<=GA<34	all	raw	0.6229	0.4888	2.03E-01
32<=GA<34	all	SEM	1.1417	0.5134	2.62E-02
GA<32	all	raw	-0.0392	0.153	7.98E-01
GA<32	all	SEM	0.106	0.161	5.08E-01
Preterm	all	raw	0.6184	0.1736	3.67E-04
Preterm	all	SEM	0.7552	0.1786	2.35E-05
Preterm	Female	SEM	0.812	0.2717	2.81E-03
Preterm	Male	SEM	0.6378	0.2394	7.73E-03
Term	all	raw	1.1993	0.0466	3.53E-146
Term	all	SEM	1.1953	0.048	3.70E-137
Term	Female	SEM	1.1516	0.0669	2.50E-66
Term	Male	SEM	1.2374	0.0686	9.46E-73

medRxiv preprint doi: https://doi.org/10.1101/2025.01.21.25320880; this version posted January 28, 2025. The copyright holder for this preprint (which was not certified by peer review) is the author/funder, who has granted medRxiv a license to display the preprint in perpetuity.
It is made available under a CC-BY 4.0 International license .

Table 3: Associations between birth length GS and birthweight in preterm- and term-born infants

Genetic Score	Gestation	Beta	SE	P
ponderal index	34<=GA<37	0.4419	0.4274	3.01E-01
ponderal index	32<=GA<34	-0.0026	0.9463	9.98E-01
ponderal index	GA<32	-0.0569	0.608	9.25E-01
ponderal index	All	0.9922	0.0894	1.20E-28
ponderal index	All (Female)	0.9679	0.1264	1.91E-14
ponderal index	All (Male)	1.0038	0.1262	1.80E-15
ponderal index	Preterm	0.2314	0.323	4.74E-01
ponderal index	Preterm (Female)	-0.2156	0.5238	6.81E-01
ponderal index	Preterm (Male)	0.5167	0.4138	2.12E-01
ponderal index	Term	1.0442	0.0913	2.57E-30
ponderal index	Term (Female)	1.0138	0.128	2.31E-15
ponderal index	Term (Male)	1.0672	0.13	2.21E-16
birth length	34<=GA<37	1.3144	0.3719	4.09E-04
birth length	32<=GA<34	0.154	0.8574	8.58E-01
birth length	GA<32	-0.723	0.522	1.75E-02
birth length	All	0.7609	0.0741	9.54E-25
birth length	All (Female)	0.7367	0.1052	2.55E-12
birth length	All (Male)	0.7855	0.1041	4.51E-14
birth length	Preterm	0.5507	0.2815	5.04E-02
birth length	Preterm (Female)	0.1777	0.4409	6.87E-01
birth length	Preterm (Male)	0.7891	0.371	3.34E-02
birth length	Term	0.7558	0.0756	1.53E-23
birth length	Term (Female)	0.7312	0.1066	6.92E-12
birth length	Term (Male)	0.7816	0.107	2.73E-13
